# NetGO 3.0: Protein Language Model Improves Large-scale Functional Annotations

**DOI:** 10.1016/j.gpb.2023.04.001

**Published:** 2023-04-17

**Authors:** Shaojun Wang, Ronghui You, Yunjia Liu, Yi Xiong, Shanfeng Zhu

**Affiliations:** 1 Institute of Science and Technology for Brain-Inspired Intelligence and MOE Frontiers Center for Brain Science, Fudan University, Shanghai 200433, China; 2 School of Life Sciences, Fudan University, Shanghai 200433, China; 3 Department of Bioinformatics and Biostatistics, Shanghai Jiao Tong University, Shanghai 200240, China; 4 Shanghai Artificial Intelligence Laboratory, Shanghai 200232, China; 5 Shanghai Qi Zhi Institute, Shanghai 200030, China; 6 MOE Key Laboratory of Computational Neuroscience and Brain-Inspired Intelligence, Fudan University, Shanghai 200433, China; 7 Shanghai Key Laboratory of Intelligent Information Processing and Shanghai Institute of Artificial Intelligence Algorithm, Fudan University, Shanghai 200433, China; 8 Zhangjiang Fudan International Innovation Center, Shanghai 200433, China

**Keywords:** Protein function prediction, Web service, Protein language model, Learning to rank, Large-scale multi-label learning

## Abstract

As one of the state-of-the-art automated function prediction (AFP) methods, NetGO 2.0 integrates multi-source information to improve the performance. However, it mainly utilizes the proteins with experimentally supported functional annotations without leveraging valuable information from a vast number of unannotated proteins. Recently, **protein language models** have been proposed to learn informative representations [*e.g.*, Evolutionary Scale Modeling (ESM)-1b embedding] from protein sequences based on self-supervision. Here, we represented each protein by ESM-1b and used logistic regression (LR) to train a new model, LR-ESM, for AFP. The experimental results showed that LR-ESM achieved comparable performance with the best-performing component of NetGO 2.0. Therefore, by incorporating LR-ESM into NetGO 2.0, we developed NetGO 3.0 to improve the performance of AFP extensively. NetGO 3.0 is freely accessible at https://dmiip.sjtu.edu.cn/ng3.0.

## Introduction

Proteins are complex molecules that play essential roles in various biological activities. To understand the underlying mechanism of an organism as a physical system, annotating the functions of proteins is a crucial task. Gene Ontology (GO) came into being in 1998 to describe varying levels of functional information on gene/RNA/protein, which contains three domains: molecular function (MF), biological process (BP), and cellular component (CC) with over 40,000 terms [1]. As of November 2022, the number of raw protein sequences is more than 230 million in Universal Protein Knowledgebase (UniProtKB), but less than 0.1% of them have experimental annotations [2]. It is thus desirable to develop high-performance computational methods to achieve automated function prediction (AFP) without costly experiments [3].

AFP is a large-scale multi-label classification problem in which multiple related GO terms are assigned to a target protein. In the last few years, several high-performance web servers have been developed for AFP, such as INGA 2.0 [4], DeepGOWeb [5], MetaGO [6], and QAUST [7]. Under the learning to rank (LTR) framework [8], GOLabeler [9], NetGO [10], and NetGO 2.0 [11] achieved a state-of-the-art performance in the recent community-wide Critical Assessment of Functional Annotation (CAFA) [3]. Specifically, NetGO 2.0 integrates protein information from different sources to encode proteins in a computer-understandable way, such as sequences, protein domains, protein–protein interaction networks, and scientific literature. However, it does not leverage valuable information from unannotated proteins (> 99.9% of all known proteins).

Recently, the idea of pre-training in natural language processing [12] has been extended to build protein language models using self-supervised learning on millions of sequences [13], [14], [15]. Most protein language models predict the masked or next amino acid within a sequence and generate protein embeddings that can generalize across downstream tasks (more details shown in File S1). Some recent studies have explored protein language models for AFP [16], [17]. However, they have a common limitation: less frequent GO terms (*e.g.*, having less than 40 annotated proteins) are excluded in the evaluation, which accounts for around 75% of total annotations in the CAFA setting. In this work, we predicted the associations between proteins and each GO term based on Evolutionary Scale Modeling (ESM)-1b embeddings, which were trained on over 250 million protein sequences [13]. Our experimental results showed that the learned representations were helpful to AFP. Therefore, we developed NetGO 3.0 by incorporating ESM-1b embeddings in order to improve the performance extensively, which highlights the predictive power of the protein language model for AFP.

## Method

### Protein language models

A challenging problem is figuring out how to represent protein sequences as fixed-length vectors that capture the realistic sequence–function relationship. Traditional methods rely on a holistic understanding of protein properties. Recently, protein language models have provided a solution that interprets protein sequences as sentences and amino acids as words to extract fundamental features of a protein with rich and systematic information. Protein language models train nonlinear neural networks with an unsupervised objective on a large-scale dataset of protein sequences [13], [14], [15], [21].

Generally, protein language models apply deep learning models such as recurrent neural networks (RNN) and Transformer to achieve statistical embeddings of proteins from tremendous sequences. UniRep represented protein sequences as fixed-length vectors by long short-term memory (LSTM) with ∼ 24 million sequences [15]. Task Assessing Protein Embeddings (TAPE) distilled protein properties from sequences by semi-supervised learning based on ResNet, LSTM, and Transformer, and then evaluated their performance on five biologically relevant tasks [21]. Moreover, a multi-task learning framework was recently proposed to incorporate structural information (*e.g.*, contact maps and structural similarity prediction tasks) to enrich protein language models [22]. Furthermore, researchers applied protein language models to study protein molecular function prediction [17]. UDSMProt put forward a task-agnostic representation for proteins and achieved good performance on protein-level prediction tasks, namely enzyme class prediction and GO prediction [16]. However, both methods should have considered less frequent GO terms.

In this study, a new component logistic regression (LR)-ESM in NetGO 3.0 was proposed to utilize ESM-1b, a 34-layer Transformer-based model trained on Universal Protein Archive (UniParc) database with 250 million protein sequences and 650 million parameters, to generate protein-level representations by average pooling across all residue-level embeddings [13].

### Implementation

NetGO 2.0 integrates seven component methods, which are Naïve, BLAST-KNN, LR-3mer, LR-InterPro, Net-KNN, LR-Text, and Seq-RNN. We replaced Seq-RNN with LR-ESM in NetGO 3.0, which makes function prediction based on a protein language model. Specifically, LR-ESM utilized ESM-1b, a 34-layer Transformer-based model trained on the UniParc database with 250 million sequences [13], to generate protein embeddings and complete prediction. As ESM-1b has a limitation of protein sequence length, we kept the first 1000 amino acids for those protein sequences longer than 1024. We then used ESM-1b to encode each amino acid as an embedding of size 1280 for a target protein. To obtain the protein-level embedding, we applied the operation of average pooling on all amino acid positions, which comprehensively collects information from sequence data alone. Finally, LR-ESM utilized protein embeddings as input to train LR classifiers and estimated the association between target proteins and each GO term.

### Benchmark datasets

As NetGO 2.0 collected the data following the setting of CAFA, we utilized the same benchmark dataset to evaluate the performance of NetGO 3.0 and the competing methods. Table S1 reports the number of proteins in the benchmark dataset.

To take advantage of the latest annotation data, we collected sequences and GO terms before January 2022 from Universal Protein (UniProt) [2], Gene Ontology Annotation (GOA) [23], and GO [1]. Similarly, we trained and updated our model on the new dataset by following the standard protocols of NetGO 2.0 [11]. Training data are all experimental annotation data before January 2020. Validation data are all experimental no-knowledge and limited-knowledge proteins annotated from January 2020 to December 2020. Testing data are all experimental no-knowledge proteins between January 2021 and December 2021. More details for the new dataset and the definition of no-knowledge and limited-knowledge proteins are listed in File S1 and Table S2.

## Results

We compared the performance of NetGO 3.0 with the competing methods on the benchmark dataset from NetGO 2.0. The performance was evaluated by area under the precision–recall curve (AUPRC) and two standard metrics in CAFA, the maximum F1-score ($F_{max}$) and the minimum semantic distance ($S_{min}$). The definitions of these three metrics are given in Section S1 of File S1.

### Performance comparison of NetGO 3.0 with its component methods and competing methods

Table 1 illustrates the test results for NetGO 3.0, NetGO 2.0, GOLabeler, DeepGOWeb, and the component methods of NetGO 3.0. Previous studies have shown that GOLabeler and NetGO 2.0 achieved top performance in CAFA3 and CAFA4, respectively [9], [11], and DeepGOWeb provided an accurate prediction for protein function by deep learning [5].

**Table 1:** **Performance comparison of NetGO 3.0 with its components and competing methods on the test set**

**Method**	$F_{max}$			**AUPRC**			$S_{min}$		
	**MF**	**BP**	**CC**	**MF**	**BP**	**CC**	**MF**	**BP**	**CC**
Naïve	0.416	0.256	0.542	0.276	0.118	0.464	5.683	14.497	6.136
BLAST-KNN	0.632	0.312	0.566	0.542	0.132	0.405	4.098	14.198	5.288
LR-3mer	0.427	0.258	0.552	0.317	0.125	0.478	5.512	14.487	6.035
LR-InterPro	0.651	0.325	0.641	0.623	0.166	0.587	4.055	14.090	5.066
Net-KNN	0.519	0.325	0.596	0.416	0.192	0.528	5.298	13.929	5.554
Seq-RNN	0.524	0.265	0.574	0.424	0.124	0.477	5.129	14.465	5.573
LR-Text	0.464	0.248	0.479	0.353	0.154	0.403	5.362	13.919	5.713
LR-ESM	0.637	0.334	0.631	0.615	0.197	0.572	3.891	13.612	5.052
DeepGOWeb	0.620	0.305	0.620	0.521	0.115	0.493	4.496	14.772	5.550
GOLabeler	0.667	0.326	0.631	0.647	0.193	0.557	3.970	13.558	5.295
NetGO 2.0	0.666	0.366	0.663	0.655	**0.269**	0.593	4.013	12.984	4.756
NetGO 3.0	**0.679**	**0.378**	**0.670**	**0.672**	0.268	**0.620**	**3.840**	**12.800**	**4.735**

*Note*: Naïve, BLAST-KNN, LR-3mer, LR-InterPro, Net-KNN, Seq-RNN, and LR-Text are component methods from NetGO 2.0. LR-ESM is a new component method which replaces Seq-RNN in NetGO 3.0. The underlined numbers imply the best performance for component methods. The bold numbers mean the best performance among competing methods. $F_{max}$, the maximum F1-score; AUPRC, area under precision–recall curve; $S_{min}$, the minimum semantic distance; MF, molecular function; BP, biological process; CC, cellular component; LR, logistic regression; KNN, k-nearest neighbors; BLAST, Basic Local Alignment Search Tool; RNN, recurrent neural networks; ESM, Evolutionary Scale Modeling; GO, Gene Ontology.

**Figure 1:**
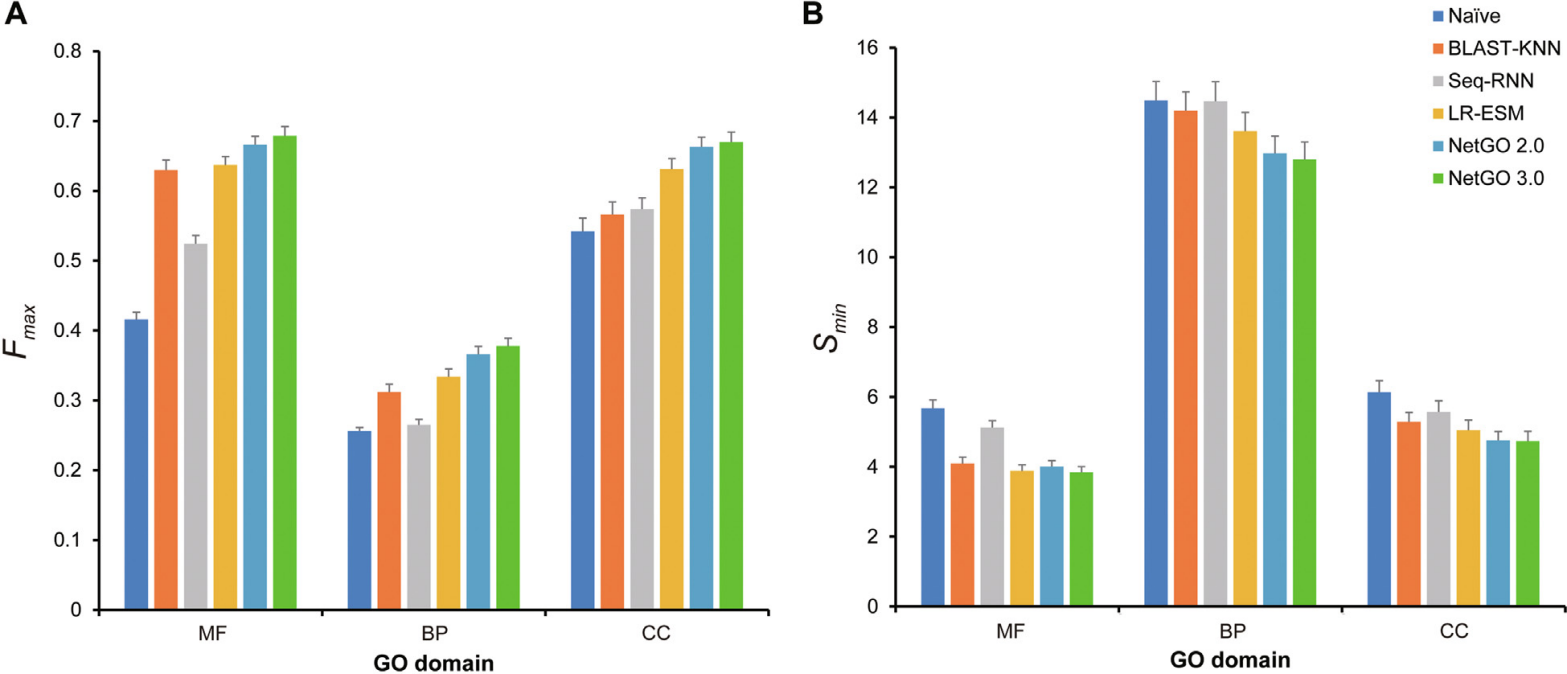
**Performance comparison on**$F_{max}$**and**$S_{min}$ The performance of Naïve, BLAST-KNN, Seq-RNN, LR-ESM, NetGO 2.0, and NetGO 3.0 on the benchmark dataset of NetGO 2.0 over three GO domains is shown. Higher values for $F_{max}$ and lower values for $S_{min}$ indicate better performance over three GO domains. The error lines denote the confidence intervals (95%) calculated by bootstrapping with 100 iterations on the test set. MF, molecular function; BP, biological process; CC, cellular component; GO, Gene Ontology; LR, logistic regression; KNN, k-nearest neighbors; BLAST, Basic Local Alignment Search Tool; RNN, recurrent neural networks; ESM, Evolutionary Scale Modeling.

We selected Naïve, BLAST-KNN, and Seq-RNN [11] from NetGO 2.0 as three baseline methods. The Naïve method annotates each pair of protein and GO term with a score that equals the probability of the term appearing in the training data. BLAST-KNN assigns a protein with GO terms based on annotations of its top BLAST hits [9]. Although the first two are component methods inherited from both NetGO and NetGO2.0, Seq-RNN is a new component of NetGO2.0, which is designed to extract the deep representation of a protein sequence [11]. As shown in Figure 1 and Table 1, LR-ESM outperformed baseline methods on all three GO domains. As a replacement for Seq-RNN, LR-ESM achieved a better performance. Specifically, in terms of $F_{max}$, LR-ESM achieved 21.6%, 31.3%, and 7.5% improvements over Seq-RNN on MF, BP, and CC, respectively, which indicates the effectiveness of ESM-1b for AFP. Moreover, LR-ESM and LR-InterPro showed comparable performance in all three GO domains (Table 1). Note that, in terms of $S_{min}$, LR-ESM outperformed all other component methods and even achieved a better performance on MF than NetGO 2.0. Therefore, it is reasonable to construct a more robust model by incorporating LR-ESM into NetGO 2.0.

Furthermore, we compared NetGO 3.0 with GOLabeler, DeepGOWeb, and NetGO 2.0, three high-performance methods in CAFA. As shown in Table 1, NetGO 3.0 achieved a more superior performance than the competing methods. In terms of $F_{max}$ and $S_{min}$, NetGO 3.0 achieved a better performance in all three GO domains. For example, NetGO 3.0 achieved the highest $F_{max}$ of 0.378 in BP, which is 16.0%, 23.9%, and 3.3% improvements over GOLabeler (0.326), DeepGOWeb (0.305), and NetGO 2.0 (0.366), respectively. The results demonstrate that NetGO 3.0 can benefit from protein language models with deep dense embeddings.

**Figure 2:**
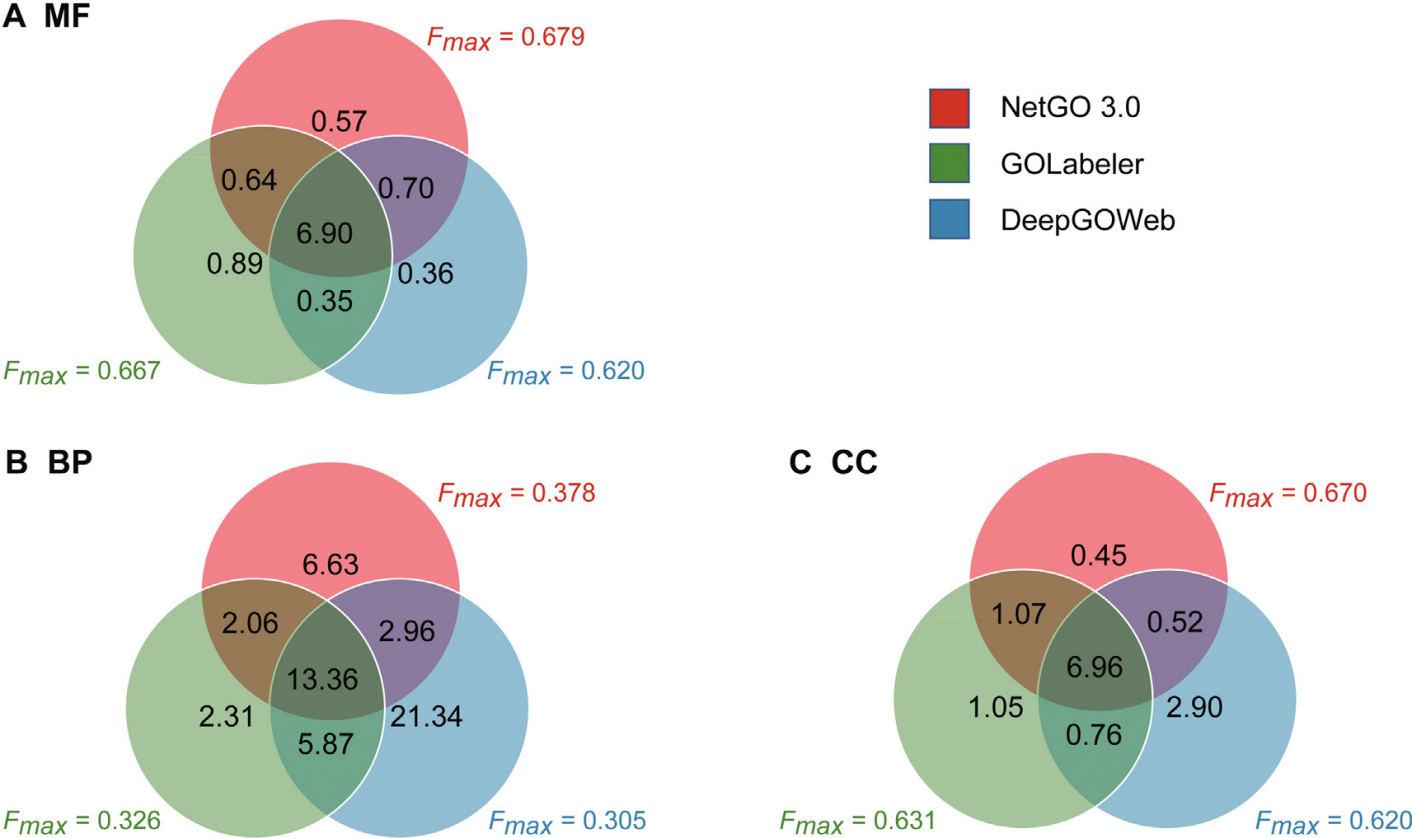
**The overlap and difference among the GO terms predicted by GOLabeler, DeepGOWeb, and NetGO 3.0** The Venn diagrams depict the overlap and difference among the GO terms predicted by GOLabeler, DeepGOWeb, and NetGO 3.0 in MF (**A**), BP (**B**), and CC (**C**), respectively. Numbers in the graph represent the average number of predicted GO terms over test proteins in three methods.

To better illustrate the strength of NetGO 3.0, we drew Venn diagrams in Figure 2 to show the overlaps and differences among the prediction results of NetGO 3.0, GOLabeler, and DeepGOWeb. There are three main findings. (1) Although each method can predict distinct GO terms, the prediction results of the three methods overlapped substantially, especially in CC. Specifically, there were 6.96 GO terms assigned to one protein on average that were predicted by all three methods in CC, which accounted for 62.5%, 70.1%, and 77.3% in the prediction results of DeepGOWeb (11.14), GOLabeler (9.84), and NetGO 3.0 (9.00), respectively. (2) DeepGOWeb predicted more GO terms but achieved lower performance than the other two methods, indicating that false-positive GO terms are common in the prediction results. For example, DeeGOWeb predicted 21.34 distinct GO terms and achieved the lowest $F_{max}$ of 0.305 in BP, which suggests that most of its predicted GO terms are incorrect. (3) Compared with MF and CC, NetGO 3.0 and GOLabeler differred significantly in predicting GO terms in BP. In terms of BP, although the 15.42 GO terms predicted by the two methods are consistent, the numbers of distinct GO terms predicted by NetGO 3.0 and GOLabeler are 9.59 and 8.18, respectively. We note that NetGO 3.0 performed better than GOLabeler in BP in terms of $F_{max}$, where NetGO 3.0 (0.378) achieved a 16.0% improvement compared with GOLabeler (0.326). It demonstrates that NetGO 3.0 is more accurate and can predict more true-positive terms for query proteins.

### Performance on specific species (humans and mice)

Species-specific analyses are helpful for researchers to study a certain species. Here, we explored the performance of different AFP methods over two model species, humans and mice. Table 2 and Table 3 showed the performance of NetGO 3.0 and NetGO 2.0, as well as the components of both methods for protein function prediction in humans and mice. We observed that all methods obtained a better prediction performance on human proteins than on mouse proteins. For example, LR-InterPro, LR-ESM, and NetGO 3.0 achieved higher AUPRC values of 0.704, 0.690, and 0.730 on human proteins in MF, whereas the three methods only achieved AUPRC values of 0.609, 0.615, and 0.620 on mouse proteins. The annotation information for different species is from different databases, which may lead to the difference. Moreover, LR-ESM again achieved a similar performance as LR-InterPro in both species, which strongly demonstrates that features extracted by ESM-1b are as robust as InterProScan among many species.

**Table 2:** **Performance comparison of NetGO 3.0 and NetGO 2.0 as well as their component methods for protein function prediction in humans**

**Method**	$F_{max}$			**AUPRC**			$S_{min}$		
	**MF**	**BP**	**CC**	**MF**	**BP**	**CC**	**MF**	**BP**	**CC**
BLAST-KNN	0.655	0.370	0.520	0.581	0.229	0.339	3.186	13.067	4.478
LR-InterPro	0.715	0.373	0.626	0.704	0.307	0.589	3.078	12.732	4.254
Net-KNN	0.598	0.358	0.592	0.565	0.243	0.536	3.882	14.304	4.756
Seq-RNN	0.596	0.291	0.585	0.523	0.184	0.536	3.850	14.729	4.401
LR-ESM	0.711	0.450	0.645	0.690	0.358	0.664	3.105	12.327	3.946
NetGO 2.0	0.715	0.441	0.673	0.725	0.401	0.630	3.018	11.917	3.566
NetGO 3.0	**0.721**	**0.481**	**0.674**	**0.730**	**0.439**	**0.670**	**2.929**	**11.451**	**3.557**

*Note*: BLAST-KNN, LR-InterPro, Net-KNN, and Seq-RNN are component methods from NetGO 2.0. LR-ESM is a new component method which replaces Seq-RNN in NetGO 3.0. The underlined numbers imply the best performance for component methods. The bold numbers mean the best performance among competing methods.

**Table 3:** **Performance comparison of NetGO 3.0 and NetGO 2.0 as well as their component methods for protein function prediction in mice**

**Method**	$F_{max}$			**AUPRC**			$S_{min}$		
	**MF**	**BP**	**CC**	**MF**	**BP**	**CC**	**MF**	**BP**	**CC**
BLAST-KNN	0.616	0.353	0.572	0.575	0.147	0.463	5.681	21.804	5.931
LR-InterPro	0.605	0.344	0.591	0.609	0.211	0.542	5.732	21.044	5.489
Net-KNN	0.408	0.341	0.566	0.253	0.199	0.502	8.080	21.309	6.002
Seq-RNN	0.520	0.265	0.537	0.373	0.106	0.462	6.787	22.795	5.993
LR-ESM	0.639	0.352	0.561	0.615	0.197	0.539	5.710	20.639	5.664
NetGO 2.0	**0.649**	0.420	0.617	0.618	0.315	0.557	5.683	19.572	5.563
NetGO 3.0	**0.649**	**0.427**	**0.620**	**0.620**	**0.316**	**0.568**	**5.583**	**19.545**	**5.034**

*Note*: BLAST-KNN, LR-InterPro, Net-KNN, and Seq-RNN are component methods from NetGO 2.0. LR-ESM is a new component method which replaces Seq-RNN in NetGO 3.0. The underlined numbers imply the best performance for component methods. The bold numbers mean the best performance among competing methods.

For human and mouse proteins, NetGO 3.0 outperformed NetGO 2.0 in all three GO domains. Specifically, NetGO 3.0 performed better than NetGO 2.0 in human BP prediction, which achieveed 9.3% and 9.5% improvements in terms of $F_{max}$ and AUPRC, respectively. Further, the results highlight the importance of source data and the effectiveness of the protein language model.

### Performance comparison over groups categorized by the number of annotations per GO term

We divided GO terms in the test dataset into three groups according to the number of annotations per GO term: 10–30, 31–100, and > 100. Table 4 showed the M-AUPRC computed in each group, where M-AUPRC is GO term-centric by averaging AUPRC on each GO term. LR-ESM outperformed other component methods in most cases, which indicates that ESM-1b embeddings are informative. Note that LR-ESM consistently ranked higher than LR-InterPro for three domains in the first group, especially for BP, which obtained a 47.8% improvement. It proves that protein embeddings are effective with such a vast amount of training data for AFP.

**Table 4:** **Performance comparison over groups categorized by the number of annotations per GO term**

**Method**	**M-AUPRC inMF**			**M-AUPRC inBP**			**M-AUPRC inCC**		
	**10–30**	**31–100**	**> 100**	**10–30**	**31–100**	**> 100**	**10–30**	**31–100**	**> 100**
BLAST-KNN	0.628	0.497	0.614	0.197	0.131	0.224	0.265	0.291	0.528
LR-InterPro	0.618	0.562	0.634	0.209	0.138	0.224	0.231	0.307	0.589
Net-KNN	0.330	0.253	0.545	0.139	0.132	0.222	0.210	0.269	0.501
Seq-RNN	0.434	0.326	0.525	0.054	0.062	0.139	0.152	0.195	0.437
LR-ESM	0.642	0.516	0.658	0.307	0.154	0.242	0.333	0.342	0.572
NetGO 2.0	0.658	0.569	0.659	0.248	0.212	0.329	0.300	0.389	0.588
NetGO 3.0	**0.675**	**0.571**	**0.665**	**0.250**	**0.213**	**0.335**	**0.386**	**0.422**	**0.636**

*Note*: BLAST-KNN, LR-InterPro, Net-KNN, and Seq-RNN are component methods from NetGO 2.0. LR-ESM is a new component method which replaces Seq-RNN in NetGO 3.0. The underlined numbers imply the best performance for component methods. The bold numbers mean the best performance among competing methods.

NetGO 3.0 achieved the best results among all the methods in every group and domain except in the first group in BP, and the improvement over NetGO 2.0 was especially significant in CC. Specifically, the advances made by NetGO 3.0 were 28.7%, 8.4%, and 8.2% for the three groups, respectively. Moreover, we collected the CC terms in the second and third layers annotated with more than ten proteins in the test set. As shown in Figure S1, NetGO 3.0 achieved a better performance on most GO terms, which strongly suggests that ESM-1b is powerful for predicting protein functions about CC.

### Performance comparison on difficult proteins

Following the CAFA setting, proteins with a BLAST identity of less than 0.6 to any protein in training data are identified as “difficult proteins” [3]. In the test set, there are 66, 85, and 70 difficult proteins in MF, BP, and CC, respectively. It is evident that methods based on homology find it hard to predict the function of difficult proteins accurately. Table 5 showed the performance of different methods in dealing with difficult proteins. As mentioned above, BLAST-KNN, a method that annotates target proteins by homology proteins, ranked last in 9 experimental settings. We found that LR-InterPro and LR-ESM were the two best-performing component methods in this scenario. For example, in terms of $S_{min}$, there is a slight difference between the two methods in three domains. LR-ESM and LR-InterPro achieved the best performance for all component methods in 6 and 3 out of 9 settings. Once again, NetGO 3.0 was proved to be the best method for predicting the function of difficult proteins.

**Table 5:** **Performance on difficult proteins**

**Method**	$F_{max}$			**AUPRC**			$S_{min}$		
	**MF**	**BP**	**CC**	**MF**	**BP**	**CC**	**MF**	**BP**	**CC**
BLAST-KNN	0.469	0.261	0.386	0.217	0.057	0.206	4.689	13.036	6.128
LR-InterPro	0.614	0.308	0.620	0.551	0.156	0.558	4.018	12.217	5.579
Net-KNN	0.555	0.319	0.591	0.308	0.184	0.528	5.341	12.564	5.543
Seq-RNN	0.486	0.223	0.560	0.312	0.097	0.430	5.264	13.427	5.946
LR-ESM	0.598	0.342	0.631	0.453	0.216	0.594	4.314	12.142	5.271
NetGO 2.0	**0.645**	0.356	0.634	0.596	0.274	0.595	4.005	11.644	4.888
NetGO 3.0	**0.654**	**0.369**	**0.668**	**0.605**	**0.276**	**0.609**	**3.969**	**11.421**	**4.782**

*Note*: BLAST-KNN, LR-InterPro, Net-KNN, and Seq-RNN are component methods from NetGO 2.0. LR-ESM is a new component method which replaces Seq-RNN in NetGO 3.0. The underlined numbers imply the best performance for component methods. The bold numbers mean the best performance among competing methods.

### Performance comparison on proteins with sequence length longer than 1000 amino acids

We performed a truncation operation for proteins longer than 1000 amino acids so that ESM-1b could generate representations for all proteins in the dataset. Focusing on the performance of each method on these long proteins helps us better understand the advantages and limitations of NetGO 3.0. There exist 21, 78, and 26 test proteins in MF, BP, and CC, respectively. Table 6 showed the prediction results of component methods, NetGO 2.0, and NetGO 3.0. We found that LR-ESM was no longer one of the best-performing component methods, which indirectly led to the worse performance of NetGO 3.0 than NetGO 2.0 in MF and BP. By comparing the performance of each method on the entire test set in Table 1, we noticed that the performance decreased for all methods except Net-KNN. This suggests that function prediction for long proteins is a challenge.

**Table 6:** **Performance comparison on proteins with sequence length longer than 1000 amino acids**

**Method**	$F_{max}$			**AUPRC**			$S_{min}$		
	**MF**	**BP**	**CC**	**MF**	**BP**	**CC**	**MF**	**BP**	**CC**
BLAST-KNN	0.514	0.272	0.549	0.271	0.119	0.465	6.349	15.176	7.072
LR-InterPro	0.595	0.312	0.638	0.407	0.111	0.603	5.389	15.454	6.253
Net-KNN	0.515	0.329	0.609	0.455	0.205	0.598	6.271	14.510	6.907
Seq-RNN	0.509	0.304	0.587	0.329	0.162	0.508	6.103	14.965	6.903
LR-ESM	0.536	0.309	0.586	0.424	0.135	0.563	5.855	15.213	6.662
NetGO 2.0	**0.587**	**0.357**	0.625	**0.497**	**0.241**	0.589	**5.312**	**13.824**	6.240
NetGO 3.0	0.577	0.348	**0.631**	0.485	0.215	**0.606**	5.452	13.947	**5.938**

*Note*: BLAST-KNN, LR-InterPro, Net-KNN, and Seq-RNN are component methods from NetGO 2.0. LR-ESM is a new component method which replaces Seq-RNN in NetGO 3.0. The underlined numbers imply the best performance for component methods. The bold numbers mean the best performance among competing methods.

Moreover, we compared the prediction performance of NetGO 2.0 and NetGO 3.0 on several unannotated proteins Q3UZV7, F1QKQ1, and Q2HX28. The sequence lengths of these three proteins are 1028, 1356, and 1409, respectively. As shown in Table S3, NetGO 2.0 achieved better AUPRC on three proteins, which indicates that the truncated sequences in long proteins are important sources of information and are critical for predicting functions. This further confirms that NetGO 3.0 needs to be improved in handling long sequences, which will be important future research work.

### Visualization of the predicted results

**Figure 3:**
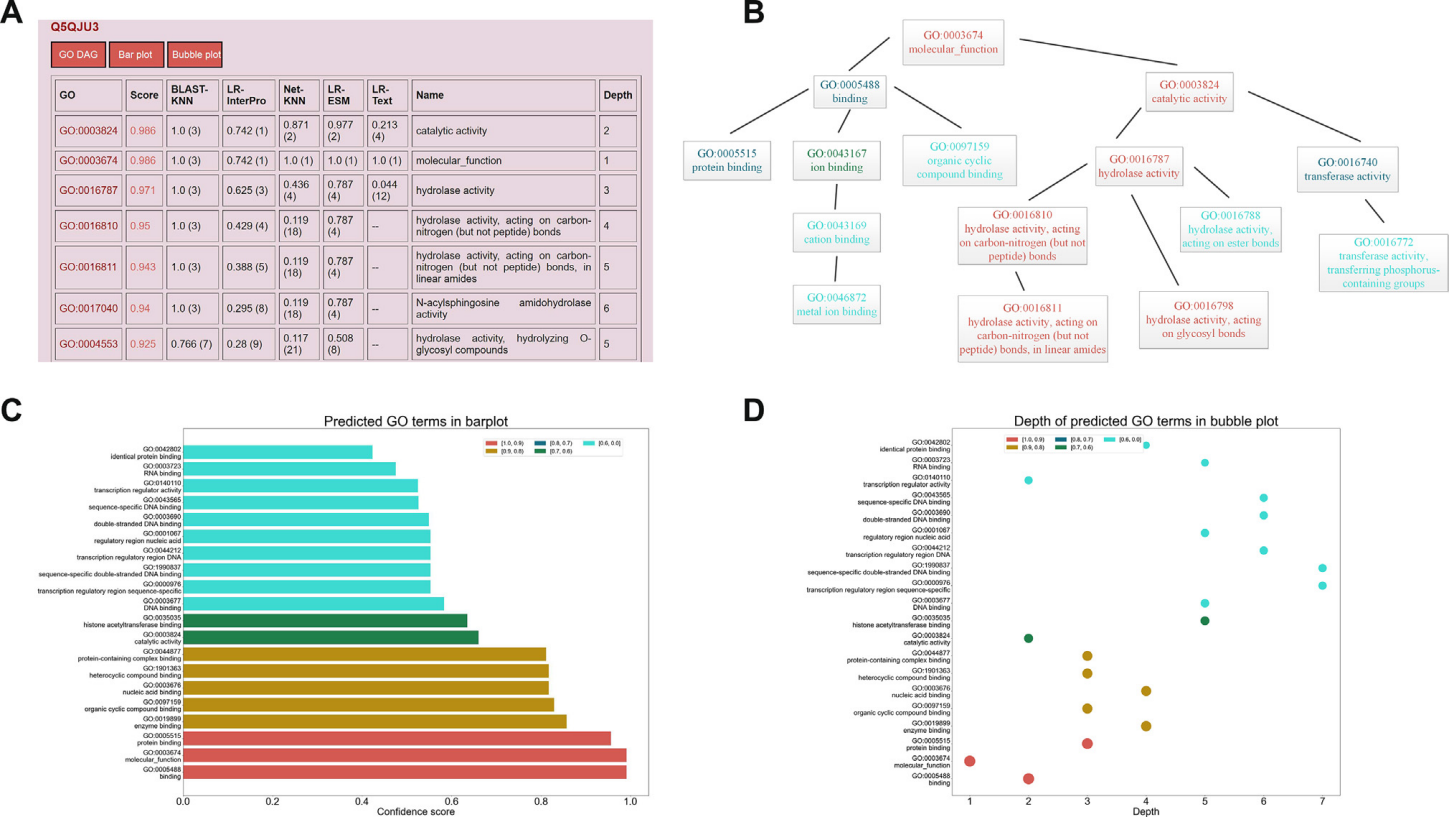
**Visualization of prediction results on the web server** **A.** Prediction result page of NetGO 3.0 website. “GO DAG”, “Bar plot”, and “Bubble plot” are the new interfaces to visualize the predicted GO terms. We also added a new column named “Depth” to show the depth of GO terms in GO analysis. **B.** The predicted GO terms and their DAGs. **C.** Bar plot showing the predicted GO terms and their confidence scores. **D.** Bubble plot showing the predicted GO terms and their depth in GO analysis. DAG, directed acyclic graph.

We presented more options to visualize the predicted GO terms to better illustrate prediction results. Compared with NetGO 2.0, the new web server offers a novel perspective to present the results, which can provide more relevant information about predicted GO terms. Figure 3 showed the new result page of NetGO 3.0, which mainly includes three ways to visualize the prediction performance. Although GO terms in top layers usually achieve a higher score and rank higher, NetGO 3.0 clarifies the depth of predicted GO terms, which allows users to find specific GO terms in bottom layers. Note that the color in the result page and node size in Figure 3D are determined by the predicted confidence score, which can help users better understand the predicted results in an original view.

### Case study

**Figure 4:**
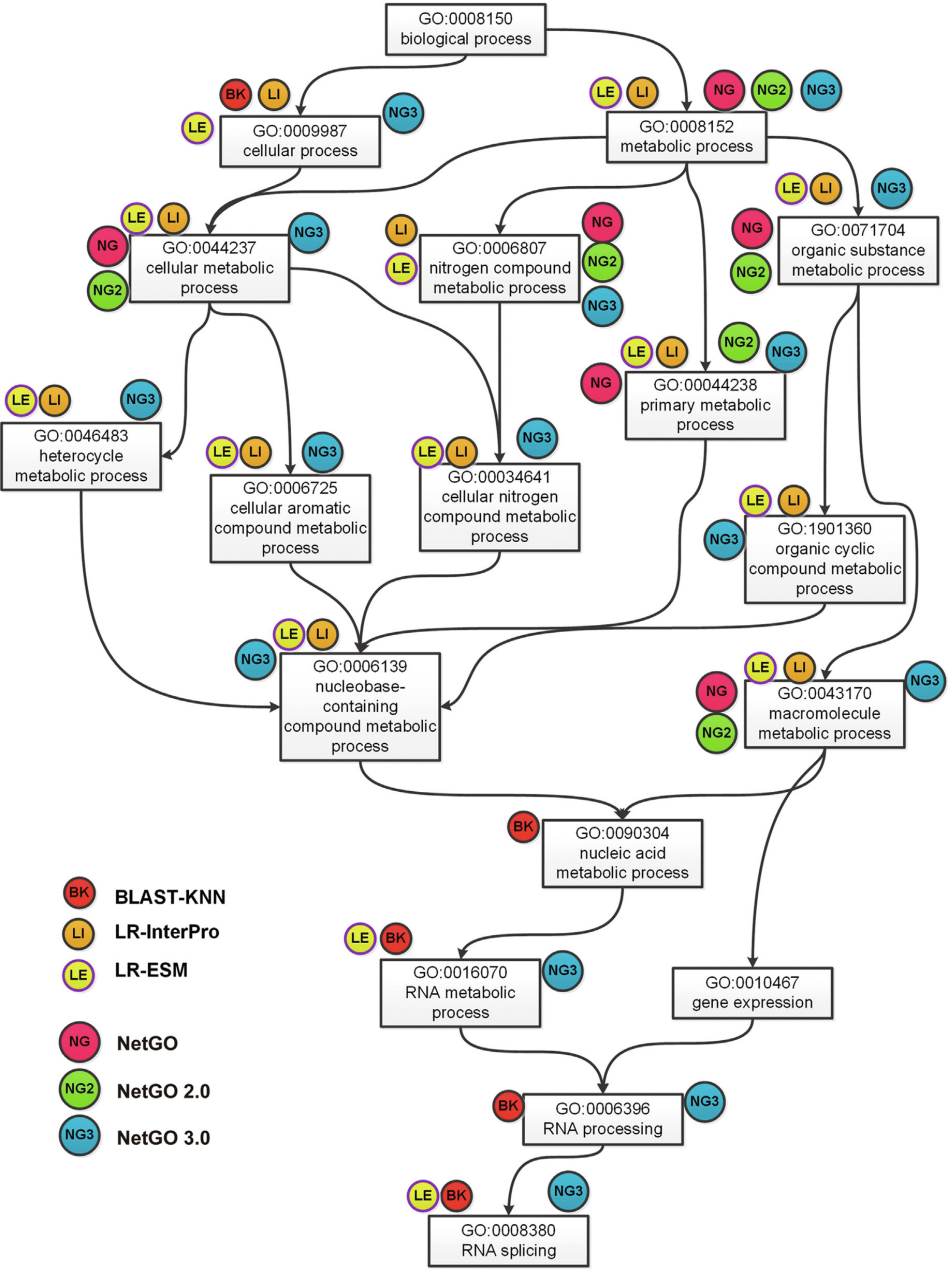
**DAG of GO terms associated with Q9FGZ9 in BP** Each GO term is attached with tags, which illustrates that the GO term is predicted correctly by corresponding methods.

Finally, we selected a specific protein as input and showed the results obtained by NetGO 3.0 and its competing methods. Ubiquitin-like protein 5 (UniProt ID: Q9FGZ9) is a difficult protein with low BLAST similarity to training proteins. Table S4 showed the 18 GO terms in BP annotated to protein Q9FGZ9. Figure 4 also depicted the directed acyclic graph (DAG) according to the relationship of 18 GO terms in GO. As shown in Table S4, BLAST-KNN failed to achieve a valid result because homology-based methods were not suitable for difficult protein function prediction. LR-InterPro and LR-ESM extracted features from raw amino acid sequences and obtained better results than BLAST-KNN. In the top 20 predicted GO terms, the number of true-positive samples achieved by LR-ESM was significantly larger than other methods, which predicted 14 correct function labels. NetGO and NetGO 2.0 predicted only six correct GO terms, which were not competitive compared to LR-ESM and NetGO 3.0. The reason for this phenomenon may be that the new component method, LR-ESM, is more robust for difficult proteins than other methods and is able to represent them more efficiently. With the support of the protein language model, NetGO 3.0 achieved 15 true GO terms out of 19 predicted ones, which successfully predicted the GO terms that NetGO and NetGO 2.0 failed to predict. Figure 4 illustrated the hierarchy of correctly predicted GO terms, indicating that NetGO 3.0 is able to predict those GO terms with less information in the deeper layers. Overall, this typical example demonstrates that the high predictive performance of NetGO 3.0 is closely related to the protein language models.

## Conclusion

We have developed NetGO 3.0 to improve the performance of large-scale AFP by incorporating a new component LR-ESM, which utilizes a protein language model to generate powerful representations of proteins. Interesting future work would be integrating protein structural information into NetGO 3.0 to enhance the performance of AFP [18], [19], [20].

## Data availability

The web server of NetGO 3.0 is freely accessible at https://dmiip.sjtu.edu.cn/ng3.0.

## Competing interests

The authors have declared no competing interests.

## CRediT authorship contribution statement

**Shaojun Wang:** Data curation, Software, Methodology, Writing – original draft. **Ronghui You:** Conceptualization, Methodology, Writing – review & editing. **Yunjia Liu:** Methodology, Visualization, Writing – review & editing. **Yi Xiong:** Resources, Writing – review & editing. **Shanfeng Zhu:** Conceptualization, Resources, Methodology, Writing – review & editing. All authors have read and approved the final manuscript.

## Supplementary material

Supplementary File S1 **Supplementary information about evaluation metrics, data collection, and performance analysis of different competing models**

Supplementary Figure S1 **The performance over M-AUPRC in CC** There are 31 GO terms in the second and third layers of the CC domain that are annotated with more than ten proteins in the test set. By checking the performance improvement of NetGO 3.0 over 2.0 on each term, the GO terms that have more than a 5% increase or decrease are shown in red or green, respectively.

Supplementary Table S1 **Summary of the benchmark dataset**

Supplementary Table S2 **Summary of the new dataset**

Supplementary Table S3 **Performance on three long proteins in BP**

Supplementary Table S4 **Predicted GO terms of Q9FGZ9 in BP by NetGO 3.0 and competing methods**

Supplementary Table S5 **Performance of different competing models**
