# Supplementary material for: NetGO 3.0: Protein Language Model Improves Large-scale Functional Annotations
Source: Genomics Proteomics Bioinformatics. 2023 Apr 17;21(2):349–58. doi: 10.1016/j.gpb.2023.04.001 (PMC10626176; doi:10.1016/j.gpb.2023.04.001)
Supplement: Supplementary File S1 — Supplementary information about evaluation metrics, data collection, and performance analysis of different competing models [file mmc1.docx]

**File S1 Supplementary information about evaluation metrics, data collection, and performance analysis of different competing models**

**Section S1 Definition of performance evaluation metrics**

The area under the precision–recall curve (AUPRC), $F_{\max}$ and $S_{\min}$ are three main metrics to evaluate the performance. AUPRC is a classical evaluation metric in machine learning especially for imbalanced classification, which punishes false positive prediction. $F_{\max}$ is an official metric of Critical Assessment of Functional Annotation (CAFA) with the following definition.

$F_{\max}=\max_{t} \left\{\frac{2*pr(t)*rc(t)}{pr(t)+rc(t)}\right\}$ (1)

where pr(t) and rc(t) are precision and recall, respectively, obtained at a cut-off value t, which is defined as follows:

$pr(t)=\frac{1}{h(t)}\sum_{j=1}^{h(t)} \frac{\sum_{i} 1(S(G_{i},P_{j})\geq t)*I(G_{i},P_{j})}{\sum_{i} 1(S(G_{i},P_{j})\geq t)}$ (2)

$rc(t)=\frac{1}{N_{T}}\sum_{j=1}^{N_{T}} \frac{\sum_{i} 1(S(G_{i},P_{j})\geq t)*I(G_{i},P_{j})}{\sum_{i} I(G_{i},P_{j})}$ (3)

where h(t) is the number of proteins with the score no smaller than t for at least one Gene Ontology (GO) term. $S(G_{i},P_{j})$ is the prediction score between protein $P_{j}$ and GO term $G_{i}$. $I(G_{i},P_{j})$ is a binary indicator, which denotes that protein $P_{j}$ has function of GO term $G_{i}$.

$S_{\min}$ means the minimum semantic distance with the definition as follows:

$ru(t)=\frac{1}{n_{e}}\sum_{i=1}^{n_{e}} \sum_{f} ic(f)*1(f\notin P_{i}(t)\wedge f\in T_{i})$ (4)

$mi(t)=\frac{1}{n_{e}}\sum_{i=1}^{n_{e}} \sum_{f} ic(f)*1(f\in P_{i}(t)\wedge f\notin T_{i})$ (5)

$S_{\min}=\min_{t} \left\{\sqrt{ru(t)^{2}+mi(t)^{2}}\right\}$ (6)

where ru(t) and mi(t) denote remaining uncertainty and misinformation at certain threshold t. In the above formulas, ic calculates the information content of GO term, given as follows:

$ic(G_{i})=\log_{2}\frac{1}{\Pr(G_{i}|parentsofG_{i}inGO)}$ (7)

where Pr(G_i_ | parents of G_i_ in GO) is the conditional probability of G_i_ given its parents of the GO structure.

**Section S2 Details of new dataset**

NetGO 3.0 collects data by following the procedures of CAFA, which mainly focus two types of proteins: no-knowledge and limited-knowledge proteins. In NetGO 3.0, no-knowledge proteins do not have any experimental annotations before 2020, while limited-knowledge proteins have at least one annotation in the other domains before 2020. Table S2 lists the numbers of proteins in the dataset.

1. Training data: all experimental annotation data before January 2020.
2. Validation data: all experimental no-knowledge and limited-knowledge proteins annotated from January 2020 to December 2020, which is listed in the column “LTR” of Table S2.
3. Testing data: all experimental no-knowledge proteins between January 2021 and December 2021.

**Section S3 Performance of** **different competing models**

As listed in Table S5, simply adding LR-ESM into NetGO 2.0 only makes a slight improvement compared with NetGO 3.0. NetGO 2.0 with LR-ESM performs better in (BP) and cellular component (CC), while NetGO 3.0 has a slight advantage in molecular function (MF). However, the difference is subtle. To reduce the model complexity, we replaced Seq-RNN with LR-ESM as a component method to develop NetGO 3.0.
