# Supplementary figures and images for: NetGO 3.0: Protein Language Model Improves Large-scale Functional Annotations
Source: Genomics Proteomics Bioinformatics. 2023 Apr 17;21(2):349–58. doi: 10.1016/j.gpb.2023.04.001 (PMC10626176; doi:10.1016/j.gpb.2023.04.001)

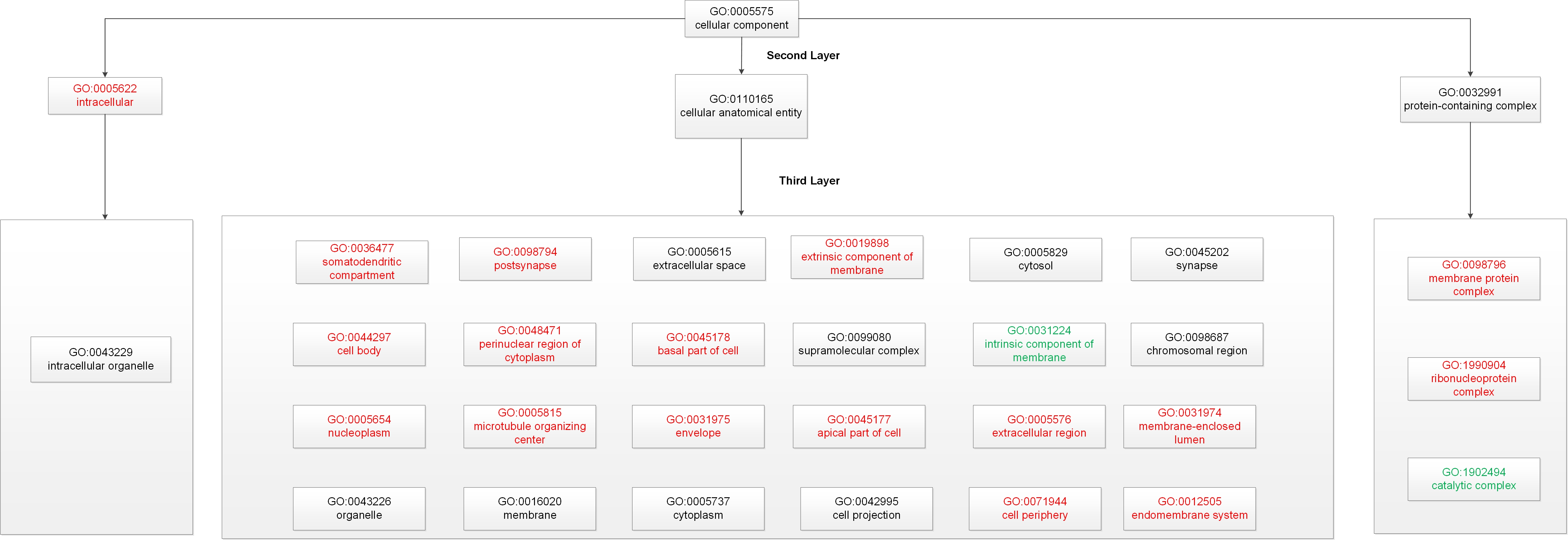

Supplement: Supplementary Figure S1 — The performance over M-AUPRC in CC There are 31 GO terms in the second and third layers of the CC domain that are annotated with more than ten proteins in the test set. By checking the performance improvement of NetGO 3.0 over 2.0 on each term, the GO terms that have more than a 5% increase or decrease are shown in red or green, respectively. [file mmc2.docx]
