# Supplementary material for: NetGO 3.0: Protein Language Model Improves Large-scale Functional Annotations
Source: Genomics Proteomics Bioinformatics. 2023 Apr 17;21(2):349–58. doi: 10.1016/j.gpb.2023.04.001 (PMC10626176; doi:10.1016/j.gpb.2023.04.001)
Supplement: Supplementary Table S2 — Summary of the new dataset [file mmc4.docx]

**Table S2 Summary of the new dataset**

|  | **Train** | | |  | **LTR** | | |  | **Test** | | |
| --- | --- | --- | --- | --- | --- | --- | --- | --- | --- | --- | --- |
|  | **MF** | **BP** | **CC** |  | **MF** | **BP** | **CC** |  | **MF** | **BP** | **CC** |
| Human | 9380 | 11,181 | 19,218 |  | 641 | 131 | 941 |  | 49 | 66 | 61 |
| Mouse | 6412 | 10,493 | 9005 |  | 273 | 224 | 189 |  | 64 | 119 | 95 |
| *Drosophila melanogaster* | 5404 | 10,045 | 7340 |  | 170 | 229 | 166 |  | 107 | 169 | 152 |
| *Arabidopsis thaliana* | 5071 | 8832 | 8469 |  | 839 | 230 | 179 |  | 73 | 129 | 127 |
| *Danio rerio* | 2743 | 11,260 | 2089 |  | 228 | 374 | 71 |  | 10 | 105 | 13 |
| Rat | 4236 | 5414 | 5041 |  | 41 | 56 | 73 |  | 17 | 47 | 31 |
| All (not only the above) | 53,680 | 87,093 | 78,458 |  | 2491 | 1654 | 1866 |  | 423 | 816 | 625 |

*Note*: LTR, learning to rank. The first six rows in the table list the data statistics on six main species and the last row records the statistics on all species (not only the above) in the dataset.
