# Supplementary material for: NetGO 3.0: Protein Language Model Improves Large-scale Functional Annotations
Source: Genomics Proteomics Bioinformatics. 2023 Apr 17;21(2):349–58. doi: 10.1016/j.gpb.2023.04.001 (PMC10626176; doi:10.1016/j.gpb.2023.04.001)
Supplement: Supplementary Table S3 — Performance on three long proteins in BP [file mmc5.docx]

**Table S3 Performance on three long proteins in BP**

|  | **AUPRC on NetGO 2.0** | **AUPRC on NetGO 3.0** | **Length** |
| --- | --- | --- | --- |
| Q3UZV7 | 0.567 | 0.471 | 1028 |
| F1QKQ1 | 0.472 | 0.265 | 1356 |
| Q2HX28 | 0.404 | 0.327 | 1409 |
