# Supplementary material for: NetGO 3.0: Protein Language Model Improves Large-scale Functional Annotations
Source: Genomics Proteomics Bioinformatics. 2023 Apr 17;21(2):349–58. doi: 10.1016/j.gpb.2023.04.001 (PMC10626176; doi:10.1016/j.gpb.2023.04.001)
Supplement: Supplementary Table S4 — Predicted GO terms of Q9FGZ9 in BP by NetGO 3.0 and competing methods [file mmc6.docx]

**Table S4 Predicted GO terms of Q9FGZ9 in BP by NetGO 3.0 and competing methods**

| **Method** | **GO terms** |
| --- | --- |
| BLAST-KNN | GO:0009987 GO:0010628 GO:0010604 GO:0010468 GO:0060255 GO:0009893 GO:0019222 GO:0048518 GO:0050789 GO:0065007 GO:0000398 GO:0000377 GO:0000375 GO:0008380 GO:0006397 GO:0016071 GO:0006396 GO:0016070 GO:0090304 |
| LR-InterPro | GO:0009987 GO:0008152 GO:0050896 GO:0044237 GO:0071704 GO:0044238 GO:0006807 GO:0043170 GO:0065007 GO:0050789 GO:0006950 GO:0044260 GO:1901564 GO:0034641 GO:0044267 GO:0019538 GO:1901360 GO:0006725 GO:0046483 |
| LR-ESM | GO:0009987 GO:0043170 GO:0071704 GO:0008152 GO:0044238 GO:0006807 GO:0044260 GO:0044237 GO:0050896 GO:0034641 GO:0046483 GO:1901360 GO:0006139 GO:0006725 GO:0043412 GO:0008380 GO:0016070 GO:0000375 GO:000377 |
| NetGO | GO:0009987 GO:0008152 GO:0044237 GO:0050896 GO:0043170 GO:0071704 GO:0006807 GO:0044238 GO:0048856 GO:0032502 GO:0032501 GO:0006950 GO:0050789 GO:0065007 GO:0007275 GO:0000003 GO:0022414 GO:0051704 GO:0042221 |
| NetGO 2.0 | GO:0009987 GO:0044237 GO:0008152 GO:0050896 GO:0071704 GO:0044238 GO:0050789 GO:0065007 GO:0043170 GO:0006807 GO:0032502 GO:0048856 GO:0006950 GO:0050794 GO:0007275 GO:0032501 GO:0003006 GO:0022414 GO:0000003 |
| NetGO 3.0 | GO:0009987 GO:0008152 GO:0044237 GO:0044238 GO:0043170 GO:0071704 GO:0006807 GO:0050896 GO:0050789 GO:0065007 GO:1901360 GO:0046483 GO:0006139 GO:0006725 GO:0034641 GO:0006950 GO:0008380 GO:0006396 GO:0016070 |
| Groud truth | GO:0044237 GO:0044238 GO:0009987 GO:0006139 GO:0046483 GO:0008150 GO:0006807 GO:0071704 GO:0006396 GO:0016070 GO:0010467 GO:0008380 GO:1901360 GO:0008152 GO:0090304 GO:0043170 GO:0034641 GO:0006725 |

*Note*: BLAST-KNN and LR-InterPro are component methods from NetGO 2.0. LR-ESM is a new component method in NetGO 3.0. Each method shows the top 20 predicted GO terms (the root term GO:0008150 is deleted). Correctly predicted GO terms are in red, and the last row shows the ground truth GO terms.
