# Supplementary material for: NetGO 3.0: Protein Language Model Improves Large-scale Functional Annotations
Source: Genomics Proteomics Bioinformatics. 2023 Apr 17;21(2):349–58. doi: 10.1016/j.gpb.2023.04.001 (PMC10626176; doi:10.1016/j.gpb.2023.04.001)
Supplement: Supplementary Table S5 — Performance of different competing models [file mmc7.docx]

**Table S5 Performance of different competing models**

| **Method** | **$F_{\max}$** | | |  | **AUPRC** | | |  | **$S_{\min}$** | | |
| --- | --- | --- | --- | --- | --- | --- | --- | --- | --- | --- | --- |
|  | **MF** | **BP** | **CC** |  | **MF** | **BP** | **CC** |  | **MF** | **BP** | **CC** |
| DeepGOWeb | 0.620 | 0.605 | 0.620 |  | 0.521 | 0.115 | 0.493 |  | 4.496 | 14.772 | 5.550 |
| GOLabeler | 0.667 | 0.326 | 0.631 |  | 0.647 | 0.193 | 0.557 |  | 3.970 | 13.558 | 5.295 |
| NetGO 2.0 | 0.666 | 0.366 | 0.663 |  | 0.655 | 0.269 | 0.593 |  | 4.013 | 12.984 | 4.756 |
| NetGO 2.0’ | **0.680** | 0.377 | **0.670** |  | **0.673** | **0.270** | 0.619 |  | 3.841 | **12.797** | **4.691** |
| NetGO 3.0 | 0.679 | **0.378** | **0.670** |  | 0.672 | 0.268 | **0.620** |  | **3.840** | 12.800 | 4.735 |

*Note*: NetGO 2.0’ denotes a model that simply adds LR-ESM into NetGO 2.0. The bold numbers mean the best performance among competing methods.
